# Herbal decoction and lumbar spine surgery in patients with lumbar disc herniation: a real-world study using linked electronic health records and claims data

**DOI:** 10.3389/fphar.2026.1824367

**Published:** 2026-06-29

**Authors:** Yoon Jae Lee, Su Won Lee, Eugene Huh, Song-Yi Kim, Minjung Park, In-Hyuk Ha

**Affiliations:** 1 Jaseng Spine and Joint Research Institute, Jaseng Medical Foundation, Seoul, Republic of Korea; 2 Department of Formulae Pharmacology, College of Korean Medicine, Gachon University, Seongnam-si, Gyeonggi-do, Republic of Korea; 3 Department of Anatomy and Acupoint, College of Korean Medicine, Gachon University, Seongnam-si, Gyeonggi-do, Republic of Korea; 4 Department of Preventive Medicine, College of Korean Medicine, Gachon University, Seongnam-si, Gyeonggi-do, Republic of Korea

**Keywords:** electronic health records, herbal medicine, insurance claims data, lumbar disc herniation, lumbar spine surgery, non-surgical treatments, real world data

## Abstract

**Introduction:**

Lumbar disc herniation (LDH) is a major cause of low back pain and may require surgical intervention when conservative or non-surgical treatment fails. In Korea, herbal medicine is commonly used as part of non-surgical care; however, its long-term effect on the risk of lumbar surgery has been difficult to assess because herbal decoctions have historically not been recorded in national insurance claims data.

**Methods:**

We conducted a retrospective cohort study by linking electronic health records (EHRs) from four Korean medical hospitals with National Health Insurance claims data. Patients newly diagnosed with LDH (ICD-10 M51) between 2016 and 2017 were included. The index date was defined as 1 year after the first hospital visit, and patients were followed until 31 July 2021. Exposure was defined as receiving herbal decoction for ≥30 days versus <30 days during the 1-year period between the entry date and index date. Patients with red-flag conditions or those who underwent lumbar surgery before the index date were excluded from the study. Propensity score matching (1:1) was performed using age, sex, insurance type, Charlson Comorbidity Index, and baseline leg pain numeric rating scale. Lumbar surgery was identified using procedure codes for discectomy, laminectomy, or spinal fusion. Kaplan-Meier analysis and Cox proportional hazards models were used to estimate the association between herbal decoction use and surgery.

**Results:**

Among 6,669 eligible patients, 2,504 received herbal decoction for ≥30 days. After propensity score matching, 2,473 patients remained in each group, with good balance across baseline covariates (standardized mean differences <0.1). The ≥30-day group showed a significantly lower cumulative incidence of lumbar surgery compared with the <30-day group (2.63% vs. 3.64%; log-rank p = 0.033). Across sequential Cox regression models, longer herbal decoction use was consistently associated with a reduced risk of lumbar surgery, hazard ratio (HR) 0.71 (95% CI 0.51–0.97).

**Discussion:**

In this real-world study linking EHR and claims data, herbal decoction use for ≥30 days was associated with a lower risk of subsequent lumbar spine surgery in patients with LDH. These findings suggest that herbal medicine may be an effective non-surgical treatment option and warrant confirmation in prospective and randomized studies.

## 1 Introduction

Lumbar disc herniation (LDH) occurs when intervertebral disc material is displaced beyond its normal boundaries, compressing adjacent nerve roots and resulting in pain, motor weakness, or sensory disturbances in the myotomal or dermatomal distribution (Kreiner et al., 2012). The condition commonly presents with low back pain (LBP) and radiating leg pain. LBP remains a leading cause of work absenteeism and reduced productivity, contributing substantially to individual and societal burdens. Management of LDH includes both surgical and non-surgical approaches. Common non-surgical treatments include pharmacological therapy, epidural steroid injections, and spinal manipulation. However, concerns regarding reoperation (Kim et al., 2019) and the development of failed back surgery syndrome, reported in a considerable proportion of surgical patients (Crites et al., 2024), have increased interest in conservative treatments that may delay or reduce the need for surgery. Acupuncture is one such modality; a systematic review and meta-analysis reported that it may provide greater pain relief than analgesics or traction (Tang et al., 2018).

In Korea, integrative medical treatments, including acupuncture, herbal medicine, pharmacopuncture, and Chuna manual therapy, are widely used as non-surgical management options for LDH. Several studies have reported their beneficial effects on pain reduction and functional improvement (Lee et al., 2022; Kim et al., 2017). However, the existing literature has largely focused on patient-reported outcomes, such as pain severity, functional status, and quality of life. Consequently, it remains unclear whether these non-surgical treatments are associated with reductions in lumbar surgery rates. In addition, long-term follow-up studies relying on patient surveys are susceptible to recall bias and loss to follow-up, limiting the accurate ascertainment of surgical outcomes. Clinically, while urgent surgery is necessary for some patients with LDH who develop progressive neurological deficits or cauda equina syndrome, most patients initially undergo conservative management before surgical decisions are made. Therefore, evaluating the long-term association between non-surgical treatments and subsequent surgery requires a focus primarily on patients who undergo conservative management.

Korea’s National Health Insurance system covers more than 97% of the population and provides a valuable source of reliable real-world data through insurance claims. Nevertheless, insurance claims data lack detailed clinical information, such as pain severity and disease status and capture only reimbursed services, making it difficult to identify treatments paid for out-of-pocket. While services such as acupuncture, moxibustion, electroacupuncture, cupping, and, since 2019, Chuna therapy for musculoskeletal conditions are reimbursed and therefore traceable in claims data; others, including herbal medicine, pharmacopuncture, and Chuna therapy prior to its reimbursement, cannot be identified without hospital-based records. In Korea, herbal medicine for LDH was historically not reimbursed by the National Health Insurance system and was therefore paid out-of-pocket, which precluded its identification in claims data. More recently, a national pilot reimbursement program (second phase, 2024–2026) has begun to cover herbal medicine prescriptions for six conditions, including LDH, reflecting growing policy interest in herbal treatment as part of conservative care (Lee et al., 2024). Nevertheless, evidence on its long-term association with surgical outcomes has remained limited.

Following legal and regulatory changes in Korea in 2020, linking hospital electronic health records (EHRs) with national insurance claims data became feasible. This advancement enabled analyses that integrate detailed clinical information from hospital records with nationwide healthcare utilization data, allowing for the long-term follow-up of surgical outcomes across different institutions while accounting for clinical severity.

Although some previous studies have used claims data to examine the association between acupuncture treatment and surgery rates for musculoskeletal conditions, evidence on the long-term impact of herbal medicine—a therapy that, until recently, remained non-reimbursed—using linked hospital EHR and national insurance claims is limited. In particular, evidence regarding the association between herbal medicine treatment and long-term surgical risk in patients with LDH who are eligible for conservative management remains scarce. Therefore, in this study, we linked EHR and prescription data from four Korean medicine hospitals with claims data from the Health Insurance Review and Assessment Service to examine whether herbal medicine treatment is associated with long-term lumbar surgery rates in patients with LDH.

## 2 Materials and methods

### 2.1 Data source

In this study, we included patients with a primary diagnosis of LDH (ICD-10 code M51) who received care at one of the four specialized Korean medicine hospitals. After receiving approval from the data review committees of each hospital (D-2021-11-001, D-2021-11-002, and D-2021-11-003), we constructed a hospital-based EHR dataset. The dataset captured demographic characteristics (age, sex, and occupation); numeric rating scale (NRS) scores for LBP and radiating leg pain at the initial visit; history of lumbar spine surgery and epidural steroid injection; and records of all reimbursed and non-reimbursed treatments received at the participating hospitals between 2015 and 31 December 2020.

All hospital data were pseudonymized prior to the analysis. Because this retrospective study used pseudonymized data, the requirement for informed consent was waived. The study protocol was approved by the Institutional Review Boards of the participating institutions (JASENG 2022-01-005, JASENG 2022-01-011, JASENG 2022-01-012, and JASENG 2022-01-013).

National Health Insurance claims data were obtained from the Health Insurance Review and Assessment Service for patients’ treatment at a Korean medical hospital with a diagnosis of M51 between 2015 and 2017. The claims dataset provided healthcare utilization records from 2015 to 31 July 2021, and all records were pseudonymized.

**FIGURE 1:**
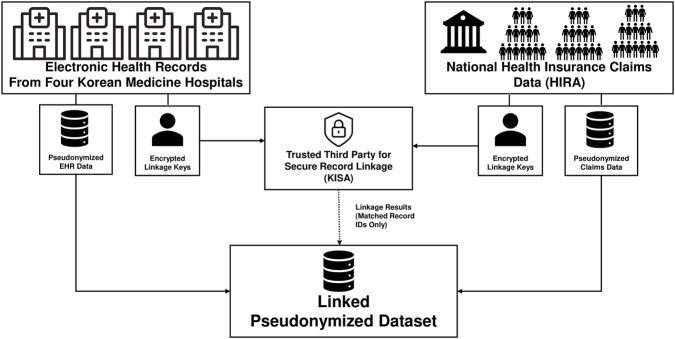
Flowchart of the data linkage process between electronic health recorrds and national health insurance claims data.

Data linkage between hospital EHR data and national claims data (approval no. C20220315881) was conducted following approval by the Health Insurance Review and Assessment Service. Each hospital and Health Insurance Review and Assessment Service independently generated encrypted linkage identifiers using the patient’s name, date of birth, and sex and transmitted them securely to a trusted third-party agency, the Korea Internet and Security Agency. The Korea Internet and Security Agency performed the record linkages and returned only the linkage results, indicating matched records, without any identifiable information. The linkage keys were used solely for the purpose of data linkages and destroyed immediately after completion. In accordance with current regulations, only successfully linked and pseudonymized records were included in the final analytic dataset. Of the 14,258 EHR records eligible for linkage, 13,437 (94.2%) were successfully linked with national claims data and constituted the final analytic dataset (Figure 1).

### 2.2 Study population

**FIGURE 2:**
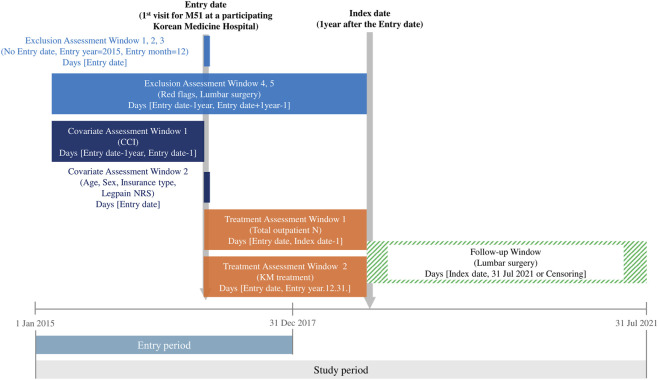
Study design and assessment windows. This figure illustrates the study timeline from 1 January 2015, to 31 July 2021. Entry Date: The first visit for lumbar disc herniation (M51) at a Korean Medicine hospital. Index Date: Defined as exactly 1 year after the Entry Date. Covariate Windows: Baseline characteristics (Age, Sex, Insurance, NRS) are assessed at the Entry Date, and comorbidities (CCI) are assessed during the preceding year. Exclusion Windows: Participants are excluded based on criteria at the Entry Date or due to red flags and surgery history within 1 year before/after the Entry Date. Treatment Windows: Frequency of outpatient visits and KM treatments are measured during the 1-year interval between the Entry Date and the Index Date. Follow-up Window: Occurs from the Index Date until 31 July 2021, or censoring, to monitor for lumbar surgery. Abbreviations: CCI: Charlson Comorbidity Index; KM: Korean Medicine; M51: Lumbar disc herniation (ICD-10 code); NRS: Numerical Rating Scale; T0 (Entry Date): First hospital visit for M51; T1 (Index Date): One year after the entry date.

The study population comprised patients with a primary diagnosis of lumbar disc herniation (ICD-10 code M51) who visited one of the four Korean medicine hospitals between 1 January 2015, and 31 December 2017. We defined the index date as 1 year after the first visit and followed patients from the index date through 31 July 2021, to ascertain lumbar spine surgery outcomes (Figure 2). This 1-year interval was established to ensure a sufficient period for assessing treatment exposure and to mitigate potential immortal time bias. By employing this landmark analysis design, we aimed to focus on patients who were candidates for long-term conservative management rather than urgent surgery. The follow-up duration from the index date to the end of the study ranged from approximately 33–55 months, depending on the patient’s entry date. The total number of outpatient visits was calculated by summing all Korean medicine and Western medicine outpatient visits associated with LBP-related diagnostic codes (ICD-10 codes: M43.0, M43.1, M47.2, M47.8, M47.9, M48.0, M51, M54.1, M54.4, M54.5, M99.3–M99.7, and S33.5–S33.7) during the 1-year exposure period.

Patients were excluded from the analysis if they met any of the following criteria: (Kreiner et al., 2012): the date of the first hospital visit could not be confirmed in the EHR dataset, (Kim et al., 2019), a red flag condition (Supplementary Table 2) was recorded between 1 year prior to the entry date and before the index date, or (Crites et al., 2024) lumbar spine surgery was performed during the same period. Because national claims data were available only from 2015 onward, the medical history of patients prior to 2015 could not be reviewed. Patients whose first visit occurred in 2015 were therefore excluded to ensure an adequate 1- year look-back period for applying the exclusion criteria and for calculating comorbidities, and only patients who had an initial visit in 2016 or 2017 were included in the final analysis (Figure 2). The Charlson Comorbidity Index (CCI) was calculated based on the ICD-10 diagnostic codes using the algorithm proposed by Quan et al. (2005), with comorbidities identified during the 1-year period preceding the entry date.

### 2.3 Intervention

The exposure of interest was defined based on the prescription of herbal decoctions at the participating hospitals between the entry and index dates. Patients were classified into the herbal treatment group if they received herbal decoctions for a cumulative total of 30 days or more (≥30 days) during the period between the entry and index dates, and into the comparison group otherwise (<30 days). This threshold was based on cumulative prescription days across the 1-year period and did not require continuous administration. The 30-day duration corresponds to the standard monthly prescription unit used for LDH treatment at the participating hospitals and is consistent with previous clinical studies of herbal medicine for LDH, in which treatment periods of approximately 30 days were the most common duration used to evaluate therapeutic effects (Guan et al., 2025; Choi et al., 2026).

### 2.4 Outcome measures

The primary outcome was lumbar spine surgery, defined as the occurrence of any of the following procedures: discectomy, laminectomy, or spinal fusion. Relevant procedure codes from the National Health Insurance claims database are provided in Supplementary Table 3. Surgical events were identified during the follow-up period, which extended from the index date to 31 July 2021.

### 2.5 Statistical analysis

Descriptive statistics were used to summarize patient demographics, comorbidities, and CCI scores. Categorical variables are reported as frequencies (n) and percentages (%), while continuous variables are expressed as means and standard deviations. Baseline differences between the herbal decoction duration groups (≥30 days vs. < 30 days) were assessed using the chi-square or Fisher’s exact test for categorical variables, and the independent t-test for continuous variables.

To minimize selection bias and control for confounding factors that could influence surgical outcomes, propensity score matching was performed. Propensity scores were estimated using logistic regression, and 1:1 nearest-neighbor matching without replacement was conducted with a caliper width of 0.1. The matching variables included sex, age, insurance type, CCI score, and leg pain NRS score. Covariate balance after matching was assessed using standardized mean differences, with a standardized mean difference greater than 0.1 indicating a meaningful imbalance.

Time-to-event outcomes were analyzed using Kaplan–Meier survival curves, and differences between groups were assessed using the log-rank test. Cox proportional hazards regression models were used to estimate the hazard ratios (HRs) and 95% confidence intervals for surgery. Sequential models were constructed to assess the robustness of the association across different levels of adjustment: Model 1 was adjusted for the group only, Model 2 was adjusted for the group and demographic variables (age and sex), Model 3 further included the CCI, and Model 4 was additionally adjusted for leg pain NRS. Finally, as a sensitivity analysis, Model 5 was constructed by further adjusting for the total number of outpatient visits to account for the potential confounding effect of healthcare utilization frequency.

All statistical analyses were conducted using the SAS Enterprise Guide version 7.1 (SAS Institute Inc., Cary, NC, United States). Statistical significance was set at a two-sided p-value of <0.05.

## 3 Result

### 3.1 Study population

A total of 13,437 patients were successfully linked between the National Health Insurance claims data and the hospital EHRs. Based on the study design, 4,939 patients whose initial visit occurred in 2015 were excluded. In addition, 460 patients with red flag conditions before the index date and 767 patients who underwent lumbar spine surgery during the exclusion period were excluded from the analysis. A further 602 patients were excluded due to incomplete data, including missing information on the date of their first visit. Ultimately, 6,669 patients were included in the final analysis.

Among the study population, 2,504 patients received herbal decoctions for 30 days or more between the entry date and the index date, whereas 4,165 patients received herbal decoctions for fewer than 30 days. After propensity score matching, 2,473 patients remained in each group, resulting in a total matched cohort of 4,946 patients (Supplementary Figure 1).

### 3.2 Baseline characteristics

Baseline characteristics before and after propensity score matching are presented in Table 1. The variables included in the matching process—sex, age, insurance type, CCI, and radiating leg pain NRS—showed standardized mean differences of <0.10 after matching, indicating adequate covariate balance between the two groups. Additional baseline characteristics are provided in Supplementary Table 1.

**TABLE 1:** Baseline characteristics of patients with lumbar disc herniation before and after propensity score matching.

Variable	Before propensity score matching			After propensity score matching		
	<30 days (n = 4,165)	≥30 days (n = 2,504)	SMD	<30 days (n = 2,473)	≥30 days (n = 2,473)	SMD
Gender, n (%)						
Male	2,310 (55.46)	1,253 (50.04)	0.11	1,263 (51.07)	1,253 (50.67)	0.01
Female	1,855 (44.54)	1,251 (49.96)	−0.11	1,210 (48.93)	1,220 (49.33)	−0.01
Age, years, n (%)						
<30	781 (18.75)	308 (12.30)	0.18	308 (12.45)	308 (12.45)	0.00
31-40	1,086 (26.07)	478 (19.09)	0.17	479 (19.37)	478 (19.33)	0.00
41-50	841 (20.19)	548 (21.88)	−0.04	549 (22.20)	548 (22.16)	0.00
51-60	752 (18.06)	540 (21.57)	−0.09	543 (21.96)	540 (21.84)	0.00
≥61	705 (16.93)	630 (25.16)	−0.20	594 (24.02)	599 (24.22)	0.00
Payer type, n (%)						
NHI	4,117 (98.85)	2,486 (99.28)	−0.04	2,459 (99.43)	2,455 (99.27)	0.02
Medicaid	48 (1.15)	18 (0.72)	0.04	14 (0.57)	18 (0.73)	−0.02
CCI, n (%)						
0	3,121 (74.93)	1,850 (73.88)	0.02	1,825 (73.8)	1,819 (73.6)	0.01
1	766 (18.39)	472 (18.85)	−0.01	473 (19.13)	472 (19.09)	0.00
2	193 (4.63)	119 (4.75)	−0.01	125 (5.05)	119 (4.81)	0.01
≥3	85 (2.04)	63 (2.52)	−0.03	50 (2.02)	63 (2.55)	−0.04
NRS (leg pain), n (%)						
<7	3,227 (77.48)	1,942 (77.56)	0.00	1,785 (72.18)	1,810 (73.19)	−0.02
≥7	938 (22.52)	562 (22.44)	0.00	688 (27.82)	663 (26.81)	0.02
NRS (LBP), n (%)						
<7	2,952 (70.88)	1,831 (73.12)	−0.05	1,914 (77.40)	1,916 (77.48)	0.00
≥7	1,213 (29.12)	673 (26.88)	0.05	559 (22.60)	557 (22.52)	0.00
Symptom on flexion, n (%)						
Yes	1,323 (31.76)	721 (28.79)	0.03	729 (29.48)	717 (28.99)	0.01
No	2,842 (68.24)	1,783 (71.21)	−0.03	1,753 (70.89)	1,765 (71.37)	−0.01
Symptom on extension, n (%)						
Yes	969 (23.27)	506 (20.21)	0.03	552 (22.32)	503 (20.34)	0.03
No	3,196 (76.73)	1,998 (79.79)	−0.03	1,930 (78.04)	1,979 (80.02)	−0.03
Symptom below the knee, n (%)						
Yes	1,384 (33.23)	860 (34.35)	0.01	827 (33.44)	855 (34.57)	0.01
No	2,781 (66.77)	1,644 (65.65)	−0.01	1,655 (66.92)	1,627 (65.79)	−0.01
Slump test, n (%)						
Positive	203 (4.87)	131 (5.23)	0.01	113 (4.57)	131 (5.3)	0.04
Negative	3,962 (95.13)	1,998 (79.79)	−0.01	2,369 (95.79)	2,351 (95.07)	−0.04

NHI, national health insurance; CCI, charlson comorbidity index; CI, confidence interval; NRS, numeric rating scale; LBP, low back pain; SMD, standardized mean difference.

### 3.3 Surgery outcomes

**FIGURE 3:**
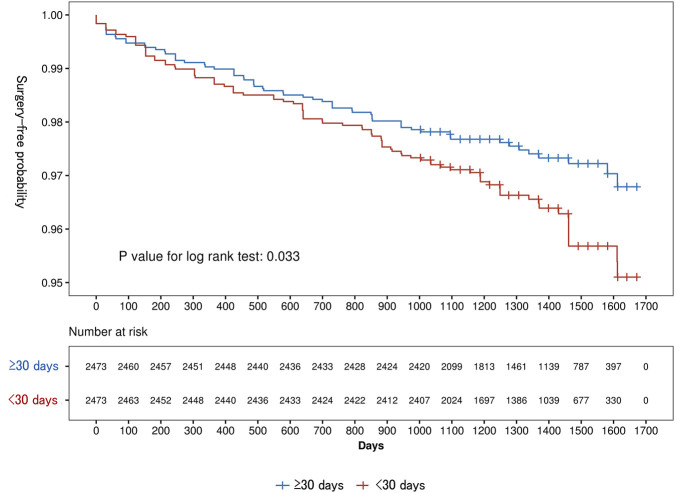
Kaplan–Meier curves for surgery-free survival after propensity score matching.

Analysis of surgery incidence using Kaplan–Meier survival curves revealed no significant difference between the two groups before propensity score matching (log-rank test, p = 0.092). After matching, however, the patients in the group that received herbal decoctions for 30 days or more showed a significantly lower incidence of lumbar spine surgery compared with those in the group that received herbal decoctions for fewer than 30 days (log-rank test, p = 0.033; Figure 3; Supplementary Table 6). Over the entire follow-up period, lumbar spine surgery occurred in 65 of 2,473 patients (2.63%) in the ≥30-day group and in 90 of 2,473 patients (3.64%) in the <30-day group, corresponding to an absolute risk difference of approximately 1.0 percentage point.

### 3.4 Cox proportional hazards analysis

Cox regression analyses revealed that patients in the ≥30-day group consistently showed a significantly lower hazard of undergoing surgery compared with those in the <30-day group, both before and after propensity score matching (HR 0.71–0.78 across models). This association remained robust after adjusting for age, sex, comorbidities, and baseline pain scores, suggesting that the duration of treatment is an independent determinant of surgical risk (Table 2). In the sensitivity analysis further adjusting for the total number of outpatient visits, this reduction in surgical risk remained significant (HR 0.60, 95% CI: 0.43-0.85; Supplementary Table 4).

**TABLE 2:** Association between duration of herbal decoction and risk of lumbar spine surgery: Multivariable Cox proportional hazards analysis.

Covariates	Before propensity score matching				After propensity score matching			
	Model 1^1^ (unadjusted)	Model 2^2^	Model 3^3^	Model 4^4^	Model 1^1^ (unadjusted)	Model 2^2^	Model 3^3^	Model 4^4^
	HR (95% CI)	HR (95% CI)	HR (95% CI)	HR (95% CI)	HR (95% CI)	HR (95% CI)	HR (95% CI)	HR (95% CI)
Group (ref. Control)	0.78 (0.58–1.04)	0.73 (0.55-0.99)*	0.74 (0.55-0.99)*	0.74 (0.55-0.99)*	0.71 (0.51-0.97)*	0.71 (0.52-0.98)*	0.71 (0.52-0.98)*	0.71 (0.52-0.98)*
Gender (ref. Male)	​	0.82 (0.62–1.09)	0.82 (0.62–1.09)	0.82 (0.62–1.09)	​	0.72 (0.52-0.99)*	0.72 (0.52-0.99)*	0.72 (0.52-0.99)*
Age (ref. < 30)								
31-40	​	1.90 (1.11-3.26)*	1.89 (1.10-3.24)*	1.89 (1.10-3.24)*	​	1.44 (0.73–2.85)	1.44 (0.73–2.85)	1.45 (0.73–2.85)
41-50	​	1.97 (1.13-3.41)*	1.94 (1.12-3.37)*	1.94 (1.12-3.37)*	​	1.57 (0.81–3.05)	1.58 (0.81–3.06)	1.58 (0.81–3.06)
51-60	​	1.97 (1.12-3.46)*	1.91 (1.09-3.37)*	1.91 (1.08-3.36)*	​	1.70 (0.88–3.28)	1.71 (0.88–3.31)	1.70 (0.88–3.31)
≥61	​	2.83 (1.66-4.85)***	2.64 (1.51-4.62)***	2.63 (1.50-4.59)***	​	2.31 (1.22-4.38)**	2.34 (1.22-4.49)*	2.36 (1.23-4.54)*
CCI (ref. 0)								
1	​	​	1.05 (0.73–1.49)	1.04 (0.73–1.49)	​	​	0.89 (0.58–1.35)	0.89 (0.59–1.36)
2	​	​	1.30 (0.74–2.30)	1.30 (0.73–2.29)	​	​	1.14 (0.59–2.21)	1.15 (0.59–2.22)
≥3	​	​	1.35 (0.62–2.97)	1.35 (0.62–2.97)	​	​	1.023 (0.369–2.834)	1.027 (0.371–2.846)
NRS_Legpain (ref. < 7)	​	​	​	1.08 (0.78–1.48)	​	​	​	0.88 (0.60–1.30)

1 Model 1: Unadjusted (crude HR for group only).

2 Model 2: Adjusted for group, demographic variables (age, sex).

3 Model 3: Adjusted for group, demographic variables (age, sex) and Charlson Comorbidity Index (CCI).

4 Model 4: Adjusted for group, demographic variables, Charlson Comorbidity Index (CCI), and leg pain NRS.

NHI, national health insurance; CCI, charlson comorbidity index; HR, hazard ratio; CI, confidence interval; NRS, numeric rating scale.

^*^ p-value <0.05.

^**^ p-value <0.01.

^***^ p-value <0.001.

## 4 Discussion

Previous studies in Korea have reported a reoperation rate of approximately 16% among patients with LDH (Kim et al., 2019), and failed back surgery syndrome has been observed in up to 20.6% of patients undergoing lumbar spine surgery (Crites et al., 2024). Given that patients with failed back surgery syndrome often experience substantially reduced quality of life, identifying effective non-surgical alternatives that may reduce the need for surgery is of considerable clinical importance. In this context, the present study examined whether herbal medicine treatment is associated with reduced long-term lumbar surgery rates in patients with LDH.

In our analysis, patients who received herbal decoctions for 30 days or more for the treatment of LDH demonstrated a significantly lower incidence of lumbar spine surgery during follow-up compared with those who received herbal decoctions for fewer than 30 days. These findings suggest a potential association between herbal medicine treatment and reduced long-term surgical rates in real-world clinical practice.

However, it is important to acknowledge that certain patients with LDH require surgery due to urgent clinical conditions. For example, progressive and significant lower limb weakness or cauda equina syndrome necessitates prompt surgical intervention (Yoon and Koch, 2021). Such acute neurological conditions cannot be reliably identified using claims data alone. To minimize the inclusion of patients with rapidly progressing disease, we excluded patients who underwent surgery soon after the initial diagnosis. In addition, lumbar spine surgery is generally considered after at least 4–6 weeks of unsuccessful conservative treatment (Yoon and Koch, 2021), a principle reflected in the Korean National Health Insurance reimbursement criteria. Considering these clinical and policy factors, we defined surgeries occurring 1 year after the initial visit as the outcomes of interest. Accordingly, the findings of this study should be interpreted primarily in the context of patients who are candidates for conservative management rather than those with clear indications for urgent surgery. Because the design required patients to remain surgery-free and free of red-flag conditions throughout the 1-year exposure ascertainment period, the matched cohort by construction represents a clinically stable population eligible for conservative management. The findings should therefore not be generalized to patients with acute neurological indications for early surgery. Patients requiring early surgery may represent a clinically distinct subgroup in which the effect of treatment exposure on subsequent surgical risk is more difficult to evaluate. By excluding such cases, this study sought to more accurately examine the association between non-surgical treatment and long-term surgical risk. This design also helped align the exposure assessment with subsequent surgical outcomes, potentially strengthening the temporal sequence and reducing the likelihood of reverse causation.

Several prior studies have examined long-term outcomes of integrative Korean medicine treatments by using surveys to assess patients’ surgical history (Lee et al., 2022; Kim et al., 2017); however, these studies often lacked control groups, were subject to recall bias, and reported high rates of loss to follow-up. In addition, large-scale claims-based studies have reported associations between acupuncture treatment and reduced surgery rates for conditions such as LBP and lumbar spinal stenosis (Koh et al., 2018; Ha et al., 2026). Yet, similar analyses focusing on herbal medicine have been scarce due to the lack of reimbursed claims data. By leveraging recent legal and regulatory changes that have enabled the linkage between hospital EHR and national insurance claims data, the present study provides real-world evidence on the long-term association between herbal medicine treatment and subsequent lumbar surgery. In this respect, this study contributes to the literature by addressing a critical gap that cannot be reliably examined using claims data alone.

In this study, the prescription of herbal decoctions was defined as the primary exposure of interest, and patients who received herbal medicines for 30 days or more were classified into the herbal treatment group. The exposure definition was established through consultation with clinicians to reflect a clinically meaningful minimum duration for evaluating the potential effects of herbal medicine treatment, and the 30 days were counted cumulatively over the 1-year period rather than requiring continuous administration. This threshold is consistent with previous clinical studies of herbal medicine for LDH, in which treatment periods of approximately 30 days were commonly used to assess therapeutic effects (Guan et al., 2025; Choi et al., 2026). Although this differs from the current pilot reimbursement criteria, which cover up to 20 days per condition each year (Lee et al., 2024), the present study was designed before these criteria were finalized. As reimbursement claims for herbal medicines accumulate through the ongoing pilot program, future studies may apply alternative exposure definitions and examine dose-response relationships across prescription-duration categories, which were not feasible in the present study.

A notable strength of this study lies in its use of hospital-based data to capture clinical severity measures, including radiating leg pain and LBP intensity, which are not available in the national insurance claims data alone. Incorporating these clinical variables allowed for propensity score matching, which accounted for baseline pain severity, a significant confounder in surgical outcome studies. To date, most claims-based analyses have been limited by the lack of detailed clinical information on pain severity, which restricts their ability to adequately control for disease severity. By integrating hospital EHR with claims data, this study addresses this limitation and provides a more clinically informed real-world analysis. Notably, the ≥30-day group initially presented with a higher mean baseline leg pain intensity (4.13 vs. 3.94) compared to <3 0-day group, despite a similar proportion of patients with severe pain (NRS ≥7). Furthermore, ≥30-day group exhibited a higher frequency of outpatient visits, with 71.2% of patients falling into the highest utilization quartiles (Q3–Q4), compared to only 36.6% in the <30-day group. Despite these factors—higher average pain severity and greater healthcare utilization—which typically increase the probability of surgical intervention, the hazard ratio decreased from 0.71 in the main analysis (Model 4) to 0.60 in the sensitivity analysis (Model 5) after additional adjustment for the total number of outpatient visits. This may indicate that the observed association is not explained by differences in healthcare-seeking behavior between the groups. However, this finding should be interpreted with caution, as the number of outpatient visits may lie on the causal pathway between herbal decoction use and surgery—whereby longer treatment leads to closer follow-up and better disease management—in which case its adjustment could represent over-adjustment. We therefore regard Model 4, without adjustment for visit frequency, as the primary analysis and treat Model 5 as an exploratory sensitivity analysis. The elevated visit frequency in ≥30-day group could also reflect the integrative nature of Korean medicine, where patients may receive frequent multi-modal treatments, such as acupuncture and Chuna manual therapy, in conjunction with herbal prescriptions.

Previous real-world studies have reported that early acupuncture treatment is associated with a reduced risk of surgery in patients with LBP (HR: 0.633) (Koh et al., 2018) and that Korean medicine treatment is associated with lower surgery rates in patients with lumbar spinal stenosis (HR: 0.821) (Ha et al., 2026). However, direct comparison with the present study findings is limited due to differences in disease characteristics, study populations, and the types of interventions evaluated.

From a mechanistic perspective, previous studies suggest that herbal medicines may attenuate intervertebral disc degeneration through multiple biological pathways, including anti-inflammatory effects, inhibition of apoptosis, maintenance of extracellular matrix homeostasis, and regulation of cellular stress responses (Melrose, 2025). As these mechanisms are derived largely from *in vitro* and animal studies (Melrose, 2025), they should be regarded as exploratory hypotheses that provide biological plausibility for the observed association, rather than as direct evidence.

However, this study had several limitations. First, several determinants of surgical decision-making could not be fully controlled. In routine clinical practice, herbal decoction is usually administered together with other Korean medicine modalities (acupuncture, Chuna manual therapy, pharmacopuncture, cupping); the observed association therefore cannot be attributed to herbal decoction alone and should be interpreted as its effect within an integrative treatment context, with the likely direction of this residual confounding being an overestimation of the herbal-specific effect. In addition, imaging severity (e.g., the degree of herniation, spinal stenosis, and Modic changes) was recorded in the source EHRs but was removed during data linkage to reduce the risk of re-identification, so residual confounding by imaging severity could not be addressed. Baseline functional status such as the Oswestry Disability Index, and physician preference, patient preference, and socioeconomic factors that influence the decision to undergo surgery were not available in the linked dataset and could not be adjusted for. Comorbidities were also assessed using a 1-year look-back period, constrained by the availability of claims data from 2015 onward, rather than the longer window often recommended for the Charlson Comorbidity Index, which may have underestimated the comorbidity burden. Second, patients were classified according to the cumulative duration of herbal decoction prescriptions, which did not require continuous administration and may therefore have grouped together patients who received intensive short-term treatment and those who accumulated prescriptions intermittently over a longer period. Herbal medicine use was also modeled as a fixed binary exposure rather than as a time-dependent covariate; although the landmark design mitigates immortal time bias by ascertaining exposure before the start of follow-up, a time-dependent analysis could more precisely capture the dynamic nature of treatment. Third, the analytic dataset retained only the duration of herbal decoction prescriptions, as prescription names were removed during the pseudonymization required for data linkage to reduce the risk of re-identification; the composition and type of individual formulations could therefore not be evaluated, particularly given that herbal medicine is frequently individualized. Adverse events and safety outcomes also could not be assessed, owing to regulatory restrictions on the use of unstructured EHR data and the scope of the linked claims dataset. More broadly, although several additional analyses—such as dose-response analyses across prescription-duration categories and a time-dependent exposure model—would have helped address these shortcomings, all analytic datasets were permanently destroyed after the predefined analysis period in accordance with the data-linkage regulations, and such supplementary analyses could no longer be performed.

Finally, the study was conducted at only four Korean medicine hospitals, whose patients may have specific healthcare-seeking behaviors, such as a strong preference for traditional medicine. By design, the matched cohort also represented a clinically stable population that remained surgery-free throughout the 1-year exposure ascertainment period and was therefore eligible for conservative management. The findings should thus not be generalized to patients with acute neurological indications for early surgery, or to the broader LDH population or non–East Asian populations.

Despite these limitations, this study used real-world data that incorporated clinical severity measures to examine the long-term association between herbal medicine treatment and subsequent lumbar surgery, which is difficult to assess using claims data alone. In this regard, our findings provide evidence that addresses an important gap in the literature concerning non-reimbursed herbal medicine treatments in routine clinical practice.

In summary, among patients with LDH, herbal medicine treatment was associated with lower lumbar surgery rates in real-world data, suggesting its potential role as a non-surgical treatment option in routine clinical practice. Further large-scale observational studies and randomized controlled trials are warranted to establish more definitive evidence on the effectiveness and safety of herbal medicines.

## Data Availability

The datasets presented in this article are not readily available due to privacy regulations and the data access policies of the Health Insurance Review and Assessment Service (HIRA), and cannot be shared publicly or upon request. Requests to access the datasets should be directed to hanihata@gmail.com.
