# Supplementary material for: Herbal decoction and lumbar spine surgery in patients with lumbar disc herniation: a real-world study using linked electronic health records and claims data
Source: Front Pharmacol. 2026 Jun 29;17:1824367. doi: 10.3389/fphar.2026.1824367 (PMC13365808; doi:10.3389/fphar.2026.1824367)
Supplement: Supplementary file 1 [file Table1.docx]

Supplementary Table 3 Baseline characteristics by covariates not used in propensity score matching

|  | Before propensity score matching | | | After propensity score matching | | |  |
| --- | --- | --- | --- | --- | --- | --- | --- |
| Variable | < 30 days (n=4,165) | ≥ 30 days (n=2,504) | SMD | < 30 days (n=2,473) | ≥ 30 days (n=2,473) | SMD |  |
| **LBP (NRS) Mean (SD)** | 5.25 (2.35) | 5.20 (2.27) | 0.02 | 5.23 (2.36) | 5.19 (2.27) | 0.02 |  |
| **Leg pain Mean (SD)** | 3.88 (3.07) | 4.13 (2.95) | -0.08 | 3.94 (3.08) | 4.13 (2.96) | -0.06 |  |
| **Days from symptom onset to visit** | | 158.34 (412.9) | 194.03 (466.2) | -0.08 | 177.4 (444.83) | 194.6 (467.35) | -0.04 |
| **Pain-free walking time (min)** | 7.16 (18.01) | 9.28 (19.88) | -0.11 | 6.91 (17.79) | 9.2 (19.82) | -0.12 |  |
| **Frequency of analgesic use** | 1.38 (4.35) | 1.43 (4.49) | -0.01 | 1.36 (4.33) | 1.43 (4.48) | -0.02 |  |
| **Frequency of nerve block** | 0.25 (0.93) | 0.29 (1.07) | -0.04 | 0.26 (0.97) | 0.29 (1.08) | -0.03 |  |
| **Presence of muscle weakness** | 147 (3.53) | 92 (3.67) | -0.01 | 95 (3.84) | 91 (3.68) | 0.01 |  |
| **Presence of sensory disturbance** | 161 (3.87) | 112 (4.47) | -0.03 | 100 (4.04) | 109 (4.41) | -0.02 |  |
| **Occupation** |  |  |  |  |  |  |  |
| Managers | 202 (4.9) | 137 (5.5) | 0.03 | 128 (5.2) | 137 (5.5) | 0.02 |  |
| Professionals & related workers | 261 (6.3) | 172 (6.9) | 0.02 | 145 (5.8) | 172 (6.9) | 0.04 |  |
| Clerks | 435 (10.4) | 277 (11.1) | 0.02 | 228 (9.2) | 274 (11.0) | 0.06 |  |
| Service & sales workers | 233 (5.6) | 156 (6.2) | 0.03 | 128 (5.2) | 155 (6.2) | 0.05 |  |
| Skilled agricultural, forestry & fishery workers | 30 (0.7) | 33 (1.3) | 0.06 | 22 (0.9) | 32 (1.3) | 0.04 |  |
| Craft & related trades workers | 62 (1.5) | 27 (1.1) | 0.04 | 36 (1.5) | 27 (1.1) | 0.03 |  |
| Plant, machine operators & assemblers | 66 (1.6) | 39 (1.6) | 0.00 | 42 (1.7) | 39 (1.6) | 0.01 |  |
| Elementary occupations | 42 (1.0) | 32 (1.3) | 0.03 | 28 (1.1) | 32 (1.3) | 0.01 |  |
| Armed forces | 69 (1.7) | 20 (0.8) | 0.08 | 31 (1.3) | 20 (0.8) | 0.04 |  |
| None (incl. homemakers/students) | 1,621 (38.9) | 1,122 (44.8) | 0.12 | 1,012 (40.8) | 1,109 (44.7) | 0.08 |  |
| **BMI**  **Mean (SD) or n(%)** | 23.16 (3.46) | 23.26 (3.16) | 0.03 | 23.33 (3.44) | 23.23 (3.15) | 0.03 |  |
| **Drinking** |  |  |  |  |  |  |  |
| Yes | 470 (11.3) | 369 (14.7) | 0.10 | 266 (10.8) | 364 (14.7) | 0.12 |  |
| No | 3,680 (88.4) | 2,121 (84.7) |  | 2,195 (88.8) | 2,095 (84.7) |  |  |
| **Smoking** |  |  |  |  |  |  |  |
| Former smoker | 30 (0.7) | 25 (1.0) | 0.03 | 19 (0.8) | 25 (1.0) | 0.02 |  |
| Current smoker | 256 (6.2) | 148 (5.9) | 0.01 | 125 (5.1) | 145 (5.9) | 0.04 |  |
| Non-smoker | 834 (20.0) | 673 (26.9) | 0.16 | 502 (20.3) | 661 (26.7) | 0.15 |  |
| **Total number of outpatient visits** |  |  |  |  |  |  |  |
| Q1 (0-25^th^ percentile) | 1,509 (36.9) | 247 (9.6) | 0.68 | 879 (34.5) | 244 (9.6) | 0.63 |  |
| Q2 (26-50^th^ percentile) | 1,191 (29.1) | 494 (19.2) | 0.23 | 735 (28.9) | 488 (19.2) | 0.23 |  |
| Q3 (51-75^th^ percentile) | 782 (19.1) | 842 (32.6) | -0.31 | 504 (19.8) | 834 (32.7) | -0.30 |  |
| Q4 (76-100^th^ percentile) | 607 (14.8) | 997 (38.6) | -0.56 | 429 (16.8) | 981 (38.5) | -0.50 |  |
