# Supplementary material for: Herbal decoction and lumbar spine surgery in patients with lumbar disc herniation: a real-world study using linked electronic health records and claims data
Source: Front Pharmacol. 2026 Jun 29;17:1824367. doi: 10.3389/fphar.2026.1824367 (PMC13365808; doi:10.3389/fphar.2026.1824367)
Supplement: Supplementary file 2 [file Table2.docx]

Supplementary Table 1. Red flags

| CODE | Full_name |
| --- | --- |
| C00-C97 | Malignant neoplasms |
| V01-V99 | Transport accidents |
| D16.6 | Benign neoplasm of Vertebral column |
| D32.1 | Benign neoplasm of Spinal meninges |
| D33.4 | Benign neoplasm of Spinal cord |
| M45 | Ankylosing spondylitis |
| M86 | Osteomyelitis |
| S32 | Fracture of lumbar spine and pelvis |
| G06.1 | Intraspinal abscess and granuloma |
| M46.0 | Spinal enthesopathy |
| M46.9 | Inflammatory spondylopathy, unspecified |
| M89.6 | Osteopathy after poliomyelitis |
| M90.2 | Osteopathy in other infectious diseases classified elsewhere |
| T09.3 | Injury of spinal cord, level unspecified |
| T08.1 | Fracture of spine, level unspecified, open |
| S34.1 | Other injury of lumbar spinal cord |
| S34.3 | Injury of cauda equina |
