# Supplementary material for: Herbal decoction and lumbar spine surgery in patients with lumbar disc herniation: a real-world study using linked electronic health records and claims data
Source: Front Pharmacol. 2026 Jun 29;17:1824367. doi: 10.3389/fphar.2026.1824367 (PMC13365808; doi:10.3389/fphar.2026.1824367)
Supplement: Supplementary file 4 [file Table3.docx]

Supplementary Table 2. Procedure Codes and Names for Lumbar Surgery

| CODE | Full_name |
| --- | --- |
| N1493 | Diskectomy(Invasive)-Lumbar Spine |
| N1494 | Diskectomy By Endoscopy |
| N1499 | Laminectomy, Lumbar Spine |
| N2499 | Laminectomy, Lumbar Spine |
| N0466 | Arthrodesis of Spine-Lumbar Spine-Anterior Technique |
| N1466 | Arthrodesis of Spine-Lumbar Spine-Anterior Technique |
| N0469 | Arthrodesis of Spine-Lumbar Spine-Posterior Technique |
| N1469 | Arthrodesis of Spine-Lumbar Spine-Posterior Technique |
| N2470' | Posterior Lumbar Interbody Fusion |
| N1460 | Posterior Lumbar Interbody Fusion |
