# Supplementary material for: Herbal decoction and lumbar spine surgery in patients with lumbar disc herniation: a real-world study using linked electronic health records and claims data
Source: Front Pharmacol. 2026 Jun 29;17:1824367. doi: 10.3389/fphar.2026.1824367 (PMC13365808; doi:10.3389/fphar.2026.1824367)
Supplement: Supplementary file 5 [file Table4.docx]

| Covariates | Before Propensity Score Matching | After Propensity Score Matching |
| --- | --- | --- |
|  | Model 5 | Model 5 |
|  | HR (95% CI) | HR (95% CI) |
| **Group (ref. Group1)** | 0.62 (0.45-0.85) ** | 0.60 (0.43-0.85) ** |
| **Gender (ref. Male)** | 0.81 (0.61-1.07) | 0.70 (0.51-0.97) * |
| **Age (ref. < 30)** |  |  |
| 31-40 | 1.85 (1.08-3.18) * | 1.42 (0.72-2.80) |
| 41-50 | 1.89 (1.09-3.29) * | 1.56 (0.80-3.02) |
| 51-60 | 1.85 (1.05-3.26) * | 1.68 (0.86-3.26) |
| ≥ 61 | 2.39 (1.36-4.18) ** | 2.18 (1.13-4.20) * |
| **CCI (ref. 0)** |  |  |
| 1 | 1.01 (0.71-1.44) | 0.85 (0.56-1.30) |
| 2 | 1.21 (0.68-2.14) | 1.07 (0.55-2.08) |
| ≥ 3 | 1.32 (0.60-2.90) | 1.02 (0.37-2.82) |
| **NRS_Legpain (ref. < 7)** | 1.02 (0.74-1.41) | 0.84 (0.57-1.24) |
| **Total number of outpatient visits (ref. Q1)** |  |  |
| Q2 (26-50^th^ percentile) | 1.67 (1.10-2.54) * | 1.34 (0.81-2.21) |
| Q3 (51-75^th^ percentile) | 1.39 (0.88-2.18) | 1.12 (0.66-1.91) |
| Q4 (76-100^th^ percentile) | 2.37 (1.55-3.63) *** | 2.15 (1.32-3.49) ** |

Supplementary Table 5 Sensitivity analysis for the association between duration of herbal decoction duration and lumbar spine surgery risk, adjusted for the total number of outpatient visits

Model 5: Adjusted for healthcare utilization, demographic variables, Charlson Comorbidity Index (CCI), leg pain NRS and total number of outpatient visits

NHI, National Health Insurance; CCI, Charlson Comorbidity Index; HR, hazard ratio; CI, confidence interval; NRS, Numeric Rating Scale

*p-value < 0.05, **p-value < 0.01, ***p-value < 0.001
