# Supplementary material for: Herbal decoction and lumbar spine surgery in patients with lumbar disc herniation: a real-world study using linked electronic health records and claims data
Source: Front Pharmacol. 2026 Jun 29;17:1824367. doi: 10.3389/fphar.2026.1824367 (PMC13365808; doi:10.3389/fphar.2026.1824367)
Supplement: Supplementary file 6 [file Table6.docx]

| Treatment | Group | Total | Event | Censored | P value for Log-rank test |
| --- | --- | --- | --- | --- | --- |
| Before Propensity Score Matching | < 30 days | 4,165 | 139 | 4,026 | 0.092 |
|  | ≥ 30 days | 2,504 | 66 | 2,438 |  |
|  | Total | 6,669 | 205 | 6,464 |  |
| After Propensity Score Matching | < 30 days | 2,473 | 90 | 2,408 | 0.033 |
|  | ≥ 30 days | 2,473 | 65 | 2,408 |  |
|  | Total | 4,946 | 155 | 4,791 |  |

Supplementary Table 4. Log-rank test results for surgery according to duration of herbal decoction before and after propensity score matching
